# Immunohistochemical detection of ALK protein identifies *APC* mutated medulloblastoma and differentiates the WNT-activated medulloblastoma from other types of posterior fossa childhood tumors

**DOI:** 10.1007/s10014-018-0331-2

**Published:** 2018-12-06

**Authors:** Maria Łastowska, Joanna Trubicka, Agnieszka Karkucińska-Więckowska, Magdalena Kaleta, Magdalena Tarasińska, Marta Perek-Polnik, Anna Antonina Sobocińska, Bożenna Dembowska-Bagińska, Wiesława Grajkowska, Ewa Matyja

**Affiliations:** 1 Department of Pathology, The Children’s Memorial Health Institute, Av. Dzieci Polskich 20, 04-730 Warsaw, Poland; 2 Department of Experimental and Clinical Pathology, Mossakowski Medical Research Centre, Polish Academy of Sciences, A. Pawińskiego 5 Street, 02-106 Warsaw, Poland; 3 Clinic of Oncology, The Children’s Memorial Health Institute, Av. Dzieci Polskich 20, 04-730 Warsaw, Poland

**Keywords:** ALK, APC, WNT-activated medulloblastoma, Immunohistochemistry

## Abstract

Expression of the *ALK* gene strongly correlates with the WNT-activated medulloblastomas, which are routinely identified by detection of *CTNNB1* mutation. However, some tumors have mutations in other than *CTNNB1* genes. Therefore, we investigated if ALK expression may identify WNT-activated tumors without *CTNNB1* mutation. In addition, we examined if ALK expression may differentiate WNT-activated medulloblastoma from other malignant posterior fossa tumors. ALK expression was analyzed using immunohistochemistry (clone D5F3) in 70 patients with posterior fossa tumours. Among 55 medulloblastomas, 6 tumors showed ALK expression in > 50% of tumor cells. In one tumor, with ALK positive reaction, negative nuclear reaction against β-catenin and the lack of *CTNNB1* mutation, next generation sequencing revealed a presence of pathogenic variant c.3366_3369del in the *APC* gene, with homozygous deletion leading to inactivation of both copies in tumor cells. MLPA analysis displayed the presence of chromosome 6 monosomy, therefore, confirming the WNT type of this tumor. All analyzed 19 anaplastic ependymomas, 4 choroid plexus carcinomas and 2 atypical teratoid rhabdoid tumors were immunonegative for ALK expression. Therefore, we propose, that immunohistochemical detection of ALK protein should be highly recommended in routine investigation, in parallel to already established methods for identification and differentiation of WNT-activated medulloblastoma.

## Introduction

Medulloblastoma is the most common malignant pediatric brain tumor. Molecular studies revealed the existence of several subgroups of this disease which depend on distinctive profiles of genes expression and DNA alterations [1–3]. The four genetically defined principal subtypes: Wingless (WNT), Sonic Hedgehog (SHH), Group 3 and Group 4 medulloblastoma are now recognized as distinct biological entities and are acknowledged in the updated World Health Organization (WHO) 2016 classification of tumors of the central nervous system [4, 5].

Importantly, several independent studies confirmed correlations between the molecular groups and clinical features, including survival of patients [2, 6]. Particularly, patients with the WNT-activated tumors have a favorable outcome of disease. In consequence, the recent SIOP-Europe trial (PNET 5-MB) introduces a reduction of the radiation dose for those patients, thus potentially reducing the severe side effects of the current treatment. Therefore, identification of patients with the WNT-activated tumors is essential because it results in clinical consequences.

Currently, as indicated in the 2016 WHO classification, there are several approaches for the identification of WNT-activated tumors, including detection of *CTNNB1* mutation, immunohistochemical positive nuclear reaction against β-catenin and the presence of chromosome 6 monosomy. Nevertheless, some tumors may not be recognized by such approach due to e.g. up-regulation of the WNT pathway by presence of mutations in other than *CTNNB1* genes, namely *APC, AXIN 1* or *AXIN 2* [7–9]. In addition, multigene expression or methylation profiling may be applied for sub-grouping purposes, but these techniques are difficult to introduce in everyday diagnostic hospital practice.

We have recently described an association between *ALK* and WNT-activated tumors. Presence of inherited *ALK* variant p.M1199L was identified in this type of disease [10] but more importantly, *ALK* expression, both at the RNA and protein levels, was strongly associated with the WNT-activated type of tumors [11].

Therefore, we further investigated if expression of *ALK* at the protein level using immunohistochemistry alone may detect WNT-activated tumors, especially those with negative nuclear reaction against β-catenin. In addition, we examined if the ALK protein expression may differentiate WNT-activated medulloblastomas from other histologically misleading high grade malignancies located in the posterior fossa in pediatric cases.

## Materials and methods

### Patients and tumor material

Overall, 70 patients with infratentorial high grade tumors diagnosed in The Children’s Memorial Health Institute (CMHI) in Warsaw, Poland, were included in the analysis. 55 patients were diagnosed with medulloblastoma (WHO grade IV), 19 patients with anaplastic ependymoma (WHO grade III), 4 patients with choroid plexus carcinoma (CPC, WHO grade IV) and 2 patients with atypical teratoid rhabdoid tumors (AT/RT, WHO grade IV). All patients were treated according to the protocol of the Polish Pediatric Neurooncology Group (PPNG).

Informed consent was obtained to use tumor material according to the procedures outlined by the CMHI’s Ethical Committee.

Analysis was performed on formalin-fixed paraffin embedded (FFPE) tissue samples collected at diagnosis. All tumors were retrospectively reviewed according to a recent WHO 2016 criteria [4, 5].

### Detection of molecular subtypes of medulloblastomas

Molecular subtypes of medulloblastomas were detected, as recommended by the 2016 WHO classification:

1. WNT-activated tumors were identified by the presence of at least two features: *CTNNB1* mutation, immunohistochemical positive nuclear reaction against β-catenin (#760-4242, clone 14, Cell Margue, dilution 1.68 µg/ml) and the presence of chromosome 6 monosomy detected by multiplex ligation-dependent probe amplification (MLPA).
2. SHH-activated tumors were determined by the presence of immunohistochemical positive reaction with anti-GAB1 (Abcam, Cambridge, USA, #ab27439 and/or ab #59362, dilution 1:100) and anti-YAP1 (Santa Cruz Biotechnology, Dallas, USA, #sc-101199, dilution 1:50) antibodies, as described by Ellison et al. [12].
3. The remaining tumors that were negative for the above features were assigned as non-WNT, non-SHH tumors.

Procedures for the detection of mutations in exon 3 of *CTNNB1* and monosomy of chromosome 6 by MLPA are described elsewhere [13].

### Detection of ALK expression by immunohistochemistry

Expression of ALK protein and scoring were described previously [11]. Briefly, antibody clone D5F3 #3633 (Cell Signaling, Denver, MA, USA) was used at dilution 1:250 and antigen retrieval was performed using EnVision FLEX HIGH pH solution (DAKO) for 30 min. at 99 °C. ALK scoring was applied as the following: 0 (negative) for 0–10%, 1 + for > 10–50%, 2 + for > 50–100% of positive tumor cells. Whole preparations were scanned in Hamamatsu NanoZoomer 2.0 RS scanner at original magnification 40 ×.

### Detection of molecular defects in patient without CTNNB1 mutation and positive ALK reaction using next-generation and Sanger sequencing

Next-generation sequencing (NGS) using genomic DNA extracted from patient’s leucocytes and tumor tissue was performed on a HiSeq 1500 using TruSight One Sequencing Panel (Illumina) according to the manufacturer’s instructions. Generated reads were aligned to the hg19 reference human genome. The detected variants were annotated using Annovar [14]. Bioinformatics analysis was performed as previously described [15]. Alignments were viewed with Integrative Genomics Viewer v.2.2.79 [16]. The nomenclature of molecular variants follows the Human Genome Variation Society guidelines (HGVS, http://www.hgvs.org/mutnomen) and referral to the cDNA sequences follows the Human Gene Mutation Database (HGMD, http://www.hgmd.af.au.uk).

The presence of the *APC* variant identified by NGS was analyzed by Sanger sequencing. The PCR reactions were carried out with the following primers: APC_16F: TTTGGACAGCAGGAATGTG and APC_16R:GGTCTCTCTTCTTCTTCATGCTG. Bidirectional sequencing was performed using a 3130 genetic analyser (Applied Biosystems, Foster City, CA, USA). The sequences were determined on both DNA strands from at least two independent PCR products. The analyzed sequence fragments were compared with the *APC* cDNA (Gen-Bank RefSeq:NM_000038.5) and protein (GenBank RefSeq:NP_000029.2) sequences using Mutation Surveyor software version 3.30 (Soft Genetics, LLC, State Collage, PA, USA). Variant positions were numbered according to HGVS recommendations (with + 1 corresponding to the A of the ATG translation initiation codon in the appropriate reference sequence).

## Results

### Patients with medulloblastoma and tumors histology

The average age of 55 medulloblastoma patients at diagnosis was 8 years, range 1–16 years. 34 patients were males, 21 patients were females. The histological diagnosis of medulloblastoma was classic in 40 cases, large cell/anaplastic (LCA) in 12 cases, desmoplastic/nodular (DN) in 2 cases, medulloblastoma with extensive nodularity (MBEN) in one case.

### Detection of ALK expression by immunohistochemistry in medulloblastoma

Overall 55 medulloblastomas were analyzed by immunohistochemistry for ALK expression. 6 tumors showed positive reaction with score 2 + (> 50–100% of positive tumor cells) and one tumors with score 1 + (> 10–50% of positive tumor cells). The remaining 48 tumors showed negative reaction (score 0, 0–10% positive tumor cells).

Among six tumors with ALK score 2 + positive reaction, four tumors showed also positive nuclear reaction against β-catenin and two tumors showed only cytoplasmic immunostaining. No *CTNNB1* mutation was detected in both cases with cytoplasmic reaction only, however, both tumors showed the presence of monosomy 6 in MLPA analysis.

Therefore, ALK positive tumors with score 2 + belonged either to the WNT group (4 tumors) or could not be classified (2 tumors). They were all of classic variant, four patients were female and two patients were male, with age from 3 to 16 years at diagnosis. Two tumors were located in cerebello-pontine angle (CPA) and four tumors had cerebellar midline location.

One tumor with positive score 1 + was classified as non-WNT/non SHH tumor and all 48 negative tumors belonged to either the non-WNT/non-SHH group (44 tumors) or SHH group (4 tumors).

### Analysis of ALK immunopositive tumor without CTNNB1 mutation

**Fig. 1:**
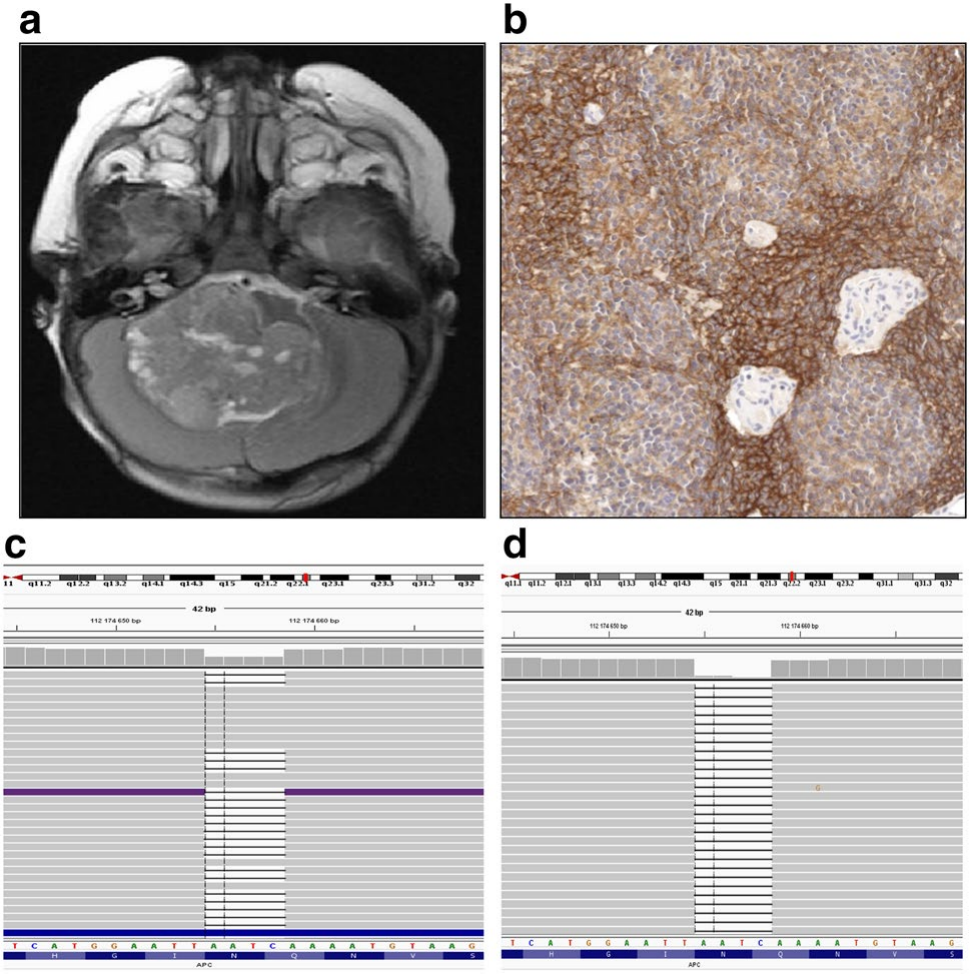
Medulloblastoma tumor with *APC* mutation. Magnetic resonance image of the tumor with cerebellar midline location (**a**); ALK immunopositive reaction present in > 80% of tumor cells (**b**); result of NGS analysis displaying the presence of the c.3366_3369del *APC* variant in heterozygous state in DNA extracted from patient’s leukocytes (**c**) and in homozygous state in tumor sample (**d**). Preparation was scanned at original magnification × 40 and digital magnification presented on image is × 20

Two ALK positive tumors with strong 2 + reaction, negative immunohistochemical nuclear reaction against β-catenin and lack of *CTNNB1* mutation could not be classified as the WNT-activated tumors according to the criteria described in the methods section. However, the presence of monosomy 6 strongly suggests that they belong to the WNT group, although the mechanism of the activation of the WNT pathway is unknown. Subsequently, we performed NGS analysis in one case, where frozen tumor and blood tissues were available, to identify possible presence of other than *CTNNB* mutations. DNA analysis revealed a presence of pathogenic variant c.3366_3369del, in *APC* gene (RefSeq RefSeq:NM_000038.5), which creates a frame shift starting at codon Asn1122. The new reading frame ends in a stop codon 3 positions downstream, p.(Asn1122Lysfs*3). Detected variant was reported as a disease causing mutation by Human Gene Mutation Database (CD920811). In silico prediction by various algorithms confirmed the pathogenic impact of identified deletion on the encoded protein. Detected variant was present in heterozygous state in patient’s leukocytes in contrast to tumor DNA where homozygous deletion leading to inactivation of both copies (loss of heterozygosity) of *APC* gene was observed (Fig. 1).

The presence of the *APC* variant identified by NGS was confirmed by Sanger sequencing. As well as *CTNNB1* mutation, *APC* mutation is also involved in up-regulation of the WNT pathway. Therefore, tumor with the presence of *APC* mutation and monosomy of chromosome 6 can be classified as the WNT type of medulloblastoma.

The patient was 12 years old male, with tumor of classic histology and cerebellar midline location, without metastases and alive 3 years since diagnosis. In addition, he suffered from polyposis coli condition, what is in line with the molecular findings.

### Detection of ALK expression by immunohistochemistry in anaplastic ependymoma, CPC and AT/RT located in the posterior fossa

**Fig. 2:**
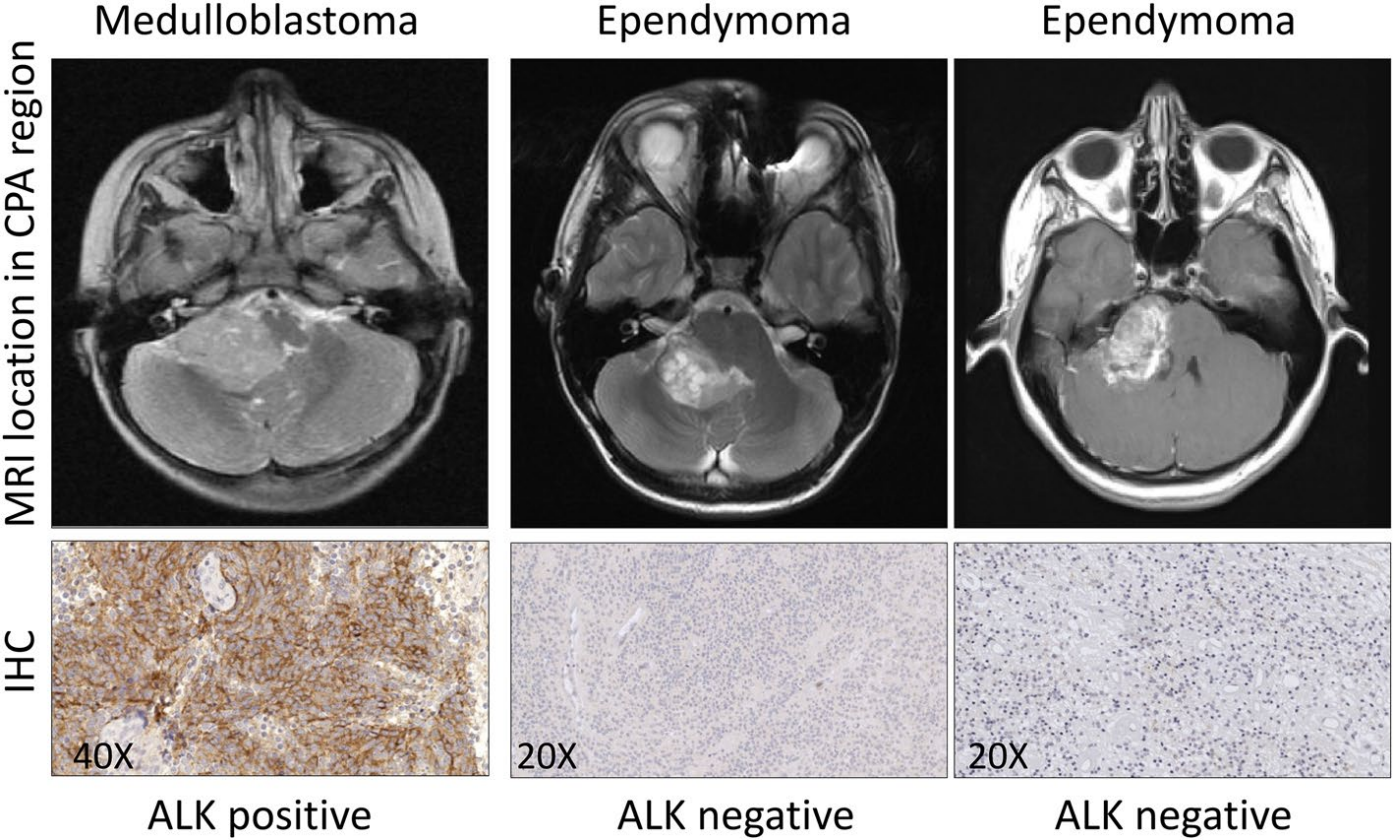
Representative ALK immunostaining in medulloblastoma and anaplastic ependymomas, all located in CPA region. Location of tumors is shown on magnetic resonance images (MRI). Immunohistochemical preparations were scanned at original magnification × 40. Digital magnification is indicated on each image

All 19 anaplastic ependymomas, 4 CPC and 2 AT/RT were negative for ALK expression. Several ependymomas were located in CPA region, similar to two cases of analyzed medulloblastomas. However, they showed dissimilar ALK immunoreactivity. Examples of ALK immunopositive medulloblastoma and ALK immunonegative ependymomas located in the CPA are presented in Fig. 2.

## Discussion

As we showed previously, a strong correlation exists between the presence of *ALK* expression and the WNT type of medulloblastoma, both at the RNA and protein levels. Interestingly, in one tumor with the presence of ALK protein expression, neither mutation in exon 3 of the *CTNNB1* gene nor nuclear β-catenin immunoreactivity were detected, but the tumor was classified to the WNT group by application of NanoString method [11]. This raises the possibility that currently recommended methods for routine identification of WNT-activated tumors may be not sufficient, without an application of multigene expression or methylation profiling.

Following this, in the present study, we extended analysis on additional 55 medulloblastomas, with a focus on ALK positive cases. Again, we detected two tumors with positive ALK immunoreactivity but without mutation in *CTNNB1* gene and negative nuclear reaction for β-catenin. NGS analysis of both tumor and blood samples in one patient revealed the presence of mutation in the *APC* gene, with homozygous deletion in tumor cells. In addition, MLPA analysis showed the presence of chromosome 6 monosomy, therefore, reassuring that this tumor belonged to the WNT group.

Mutations in the *APC* gene in medulloblastoma have been known for some time [7] and very recently, germline mutations were identified in a large series of patients with medulloblastoma using NGS analysis [17]. *APC* gene is one of the consensus medulloblastoma predisposition genes and patients develop specifically WNT-activated type of tumors. *APC* mutations were the most frequent (71%) mutations in WNT-activated tumors where the *CTNNB1* gene was not altered.

It is, therefore, possible that WNT-activated tumors can be more frequent than already detected by routine diagnostic methods. This may be related to the fact that nuclear β-catenin immunoreactivity is sometimes only focal or absent even in tumors harboring *CTNNB1* mutations [12] and other mutations activating the WNT pathway are not routinely investigated. Such cases may be classified as non-WNT/non-SHH tumors, with clinical consequences for the patient and potential impact on survival analyses. Therefore, the fact that ALK expression is positive in *APC* mutated tumor reinforces the usefulness of this marker for detection of the WNT type of medulloblastoma.

WNT-activated tumors may be located in cerebellar midline or the CPA region, as opposite to SHH tumors located usually in cerebellar hemispheres and non-WNT/non-SHH tumors characterized by cerebellar midline location.

In pediatric patients, the differential diagnosis for medulloblastomas includes other primary malignant brain tumors that arise in the posterior fossa. The most challenging malignances to be considered are: AT/RTs, CPCs and anaplastic ependymomas. These highly cellular neoplasms can show the advantage of small round poorly differentiated elements, similar to medulloblastoma. For instance, anaplastic ependymoma, located in CPA or cerebellar midline regions, may be confused with medulloblastoma, especially when the later tumor shows the presence of pseudorosette-like structures. In addition, AT/RT often includes a small blue cells population, in addition to characteristic rhabdoid phenotype, thus mimicking the large cell/anaplastic medulloblastoma. Similarly, CPC may contain variable population of poorly differentiated cells that can often be difficult to recognize solely on the basis of histology. Therefore, the identification of these lesions requires application of a wide panel of antibodies or molecular studies.

Based on our results, we indicate that ALK protein expression may easily differentiate WNT-activated medulloblastomas from other histologically similar high-grade pediatric tumors located in the posterior fossa (cerebellum/fourth ventricle/ cerebellopontine angle). Nevertheless, more cases should be examined for differentiation purposes to confirm our findings.

In summary, we propose that the immunohistochemical detection of ALK protein should be routinely investigated, in parallel to already established methods for identification of WNT-activated medulloblastomas. In case of positive ALK reaction and absence of *CTNNB1* mutation, other genes, especially *APC*, should be analyzed.
